# Measuring the sustainability of a community safety promotion network: working from the inside out 

**Published:** 2019-08

**Authors:** Dale Hanson

**Affiliations:** ^*a*^Associate Professor, Chair. International Safe Community Certifying Center, Australia.

## Abstract

**Background:**

While project sustainability is a mandatory piece of politically correct rhetoric, it is less often achieved. Mackay Safe Communities (MSC) was developed using a capacity building model that consciously attempted to design sustainability into the network. This study sought to analyze the sustainability of MSC by tracking the exchange of resources using Social Network Analysis (SNA).

**Methods:**

A snowballing methodology was used to identify the MSC and its external support network (ESN). Respondents were asked to identify the type and amount of resources they shared with other network partners.

**Results:**

The study identified 168 members of the MSC and its ESN. Thirty-five percent of relationships did not share any resources; 47% shared in-kind resources, 54% shared human resources and 15% shared financial resources. In 2004 MSC accessed an estimated 6.5 full time staff equivalents and $0.9 million Australian dollars. However, these resources were largely accessed through, and controlled by, the ESN.

**Conclusions:**

MSC was rich in social resources, but considerable in-kind, human and financial resources were accessed through its ESN. The bridging relationships that connected MSC to its ESN, more than half of which were maintained by six broker network facilitators, were the critical social asset required to access resources and thereby sustain network productivity. MWSC is an open network. Open social networks can never be totally self-sufficient. The ongoing productivity of a social network is dependent on its capacity to develop and maintain productive links with external partners.

**Keywords:**

Safe Communities, Sustainability, Evaluation

